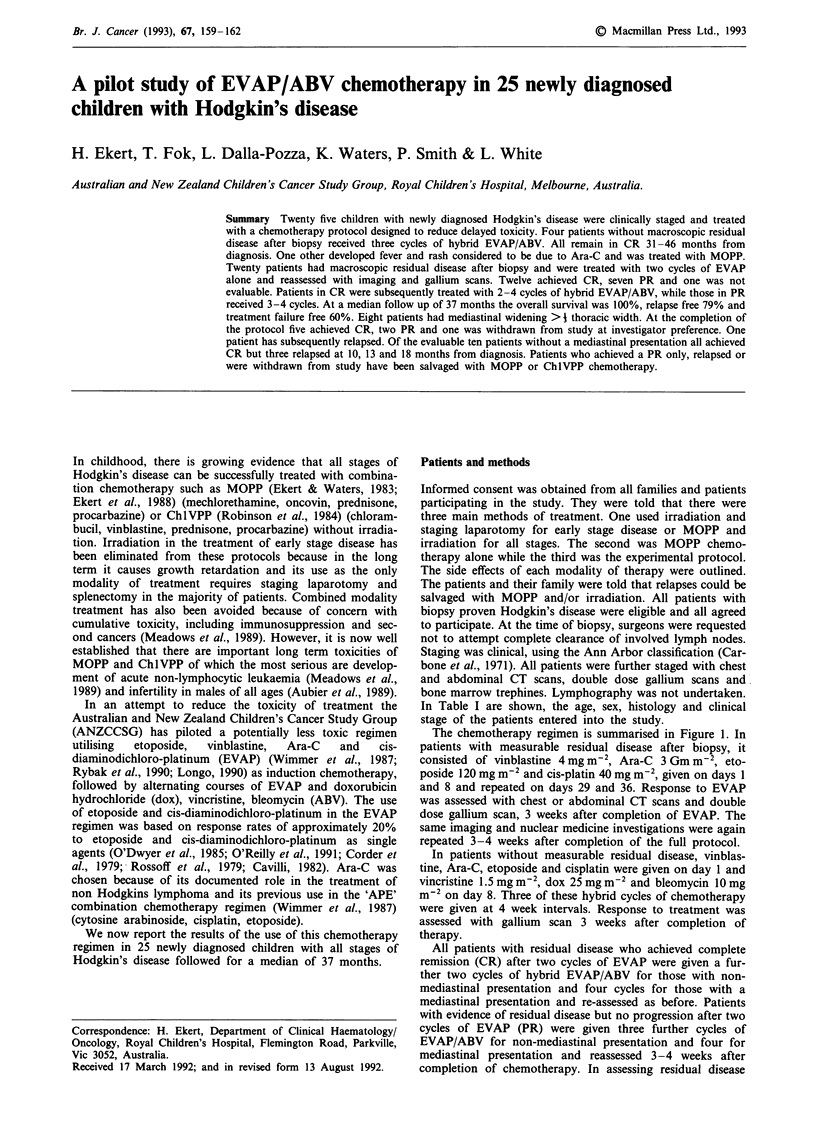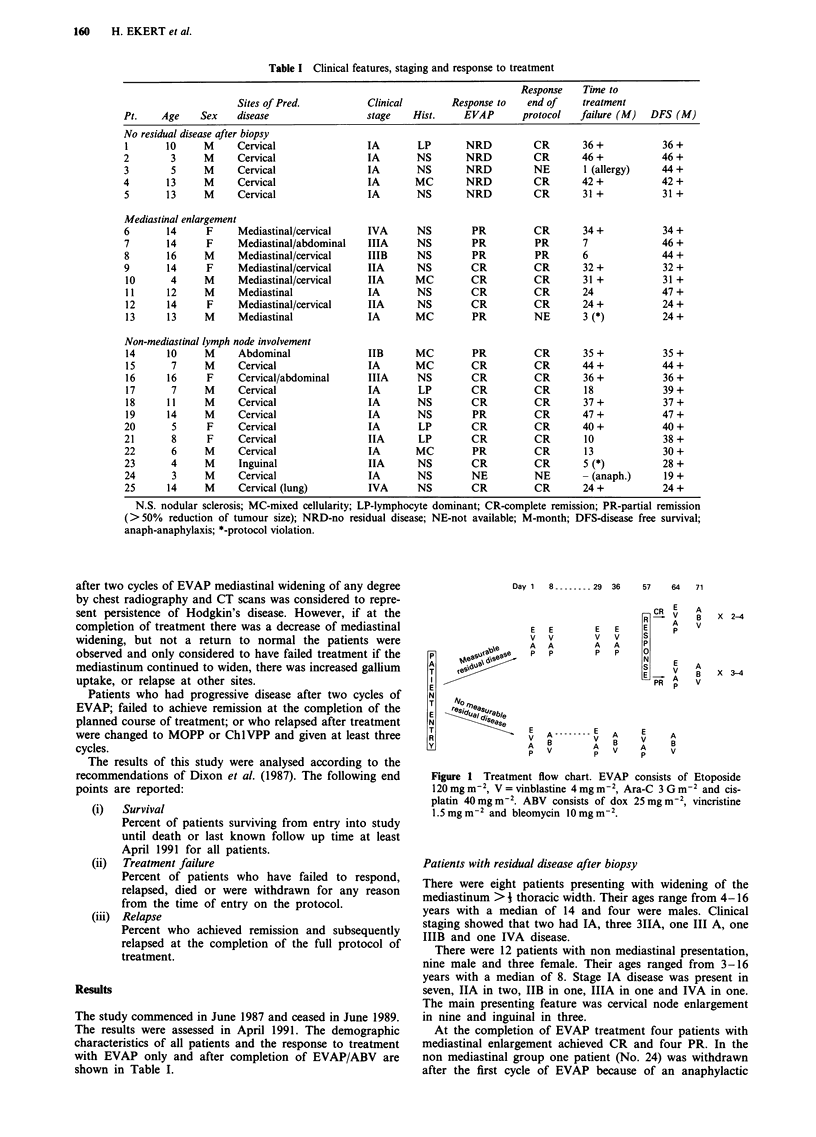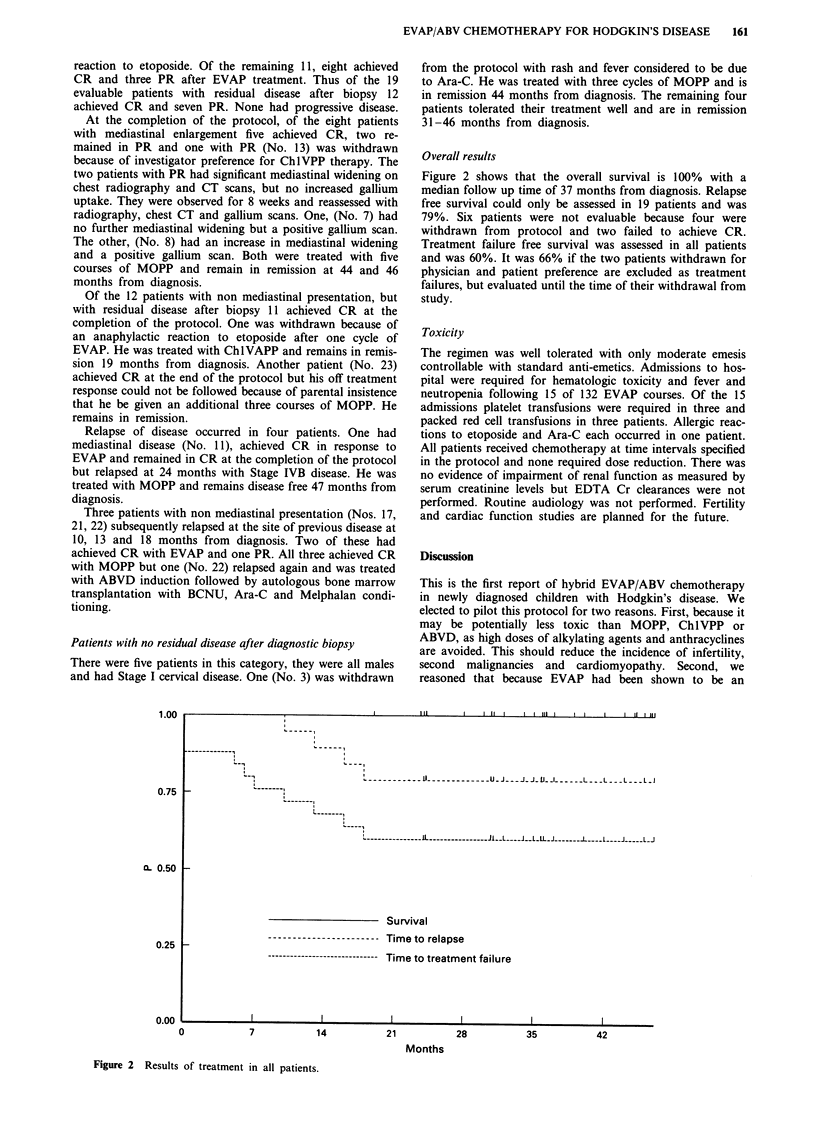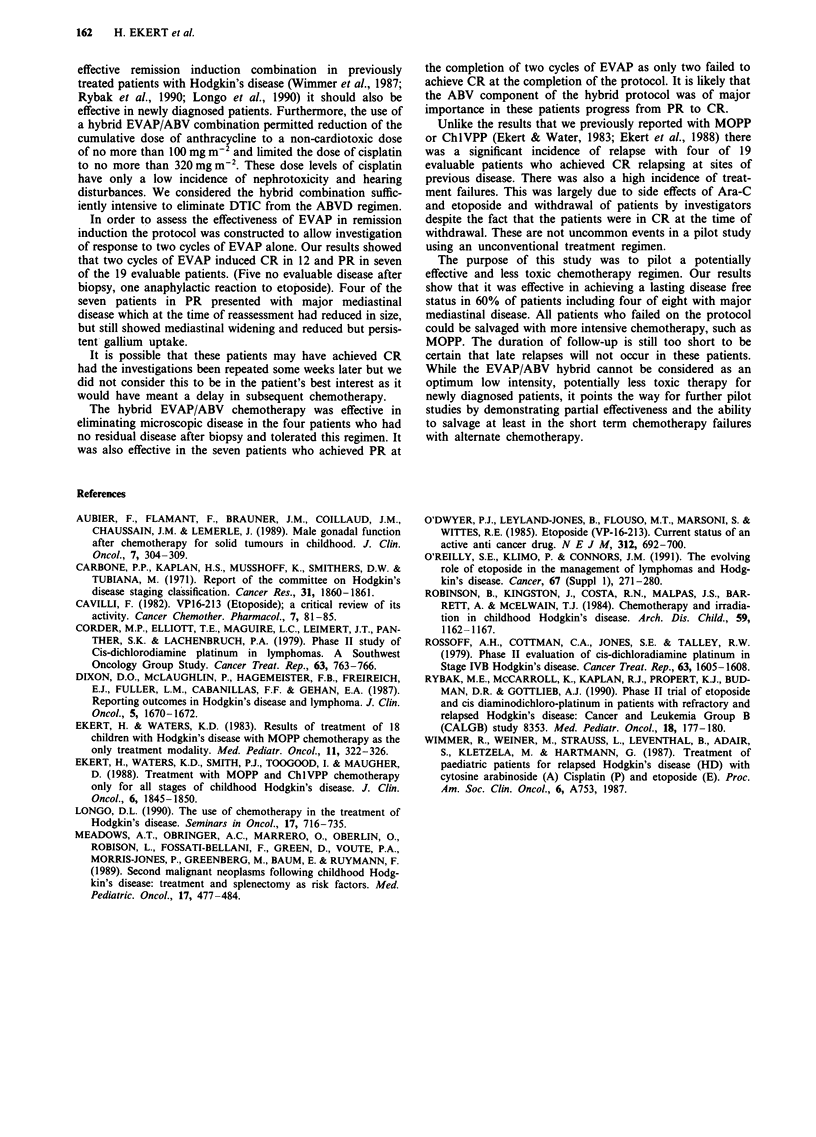# A pilot study of EVAP/ABV chemotherapy in 25 newly diagnosed children with Hodgkin's disease.

**DOI:** 10.1038/bjc.1993.28

**Published:** 1993-01

**Authors:** H. Ekert, T. Fok, L. Dalla-Pozza, K. Waters, P. Smith, L. White

**Affiliations:** Australian and New Zealand Children's Cancer Study Group, Royal Children's Hospital, Melbourne.

## Abstract

Twenty five children with newly diagnosed Hodgkin's disease were clinically staged and treated with a chemotherapy protocol designed to reduce delayed toxicity. Four patients without macroscopic residual disease after biopsy received three cycles of hybrid EVAP/ABV. All remain in CR 31-46 months from diagnosis. One other developed fever and rash considered to be due to Ara-C and was treated with MOPP. Twenty patients had macroscopic residual disease after biopsy and were treated with two cycles of EVAP alone and reassessed with imaging and gallium scans. Twelve achieved CR, seven PR and one was not evaluable. Patients in CR were subsequently treated with 2-4 cycles of hybrid EVAP/ABV, while those in PR received 3-4 cycles. At a median follow up of 37 months the overall survival was 100%, relapse free 79% and treatment failure free 60%. Eight patients had mediastinal widening > 1/3 thoracic width. At the completion of the protocol five achieved CR, two PR and one was withdrawn from study at investigator preference. One patient has subsequently relapsed. Of the evaluable ten patients without a mediastinal presentation all achieved CR but three relapsed at 10, 13 and 18 months from diagnosis. Patients who achieved a PR only, relapsed or were withdrawn from study have been salvaged with MOPP or Ch1VPP chemotherapy.


Br. J. Cancer (1993), 67, 159–162 © Macmillan Press Ltd., 1993

### A pilot study of EVAP/ABV chemotherapy in 25 newly diagnosed children with Hodgkin's disease

H. Ekert, T. Fok, L. Dalla-Pozza, K. Waters, P. Smith & L. White

*Australian and New Zealand Children's Cancer Study Group, Royal Children's Hospital, Melbourne, Australia.*

**Summary** Twenty five children with newly diagnosed Hodgkin's disease were clinically staged and treated with a chemotherapy protocol designed to reduce delayed toxicity. Four patients without macroscopic residual disease after biopsy received three cycles of hybrid EVAP/ABV. All remain in CR 31–46 months from diagnosis. One other developed fever and rash considered to be due to Ara-C and was treated with MOPP. Twenty patients had macroscopic residual disease after biopsy and were treated with two cycles of EVAP alone and reassessed with imaging and gallium scans. Twelve achieved CR, seven PR and one was not evaluable. Patients in CR were subsequently treated with 2–4 cycles of hybrid EVAP/ABV, while those in PR received 3–4 cycles. At a median follow up of 37 months the overall survival was 100%, relapse free 79% and treatment failure free 60%. Eight patients had mediastinal widening $>\frac{1}{3}$ thoracic width. At the completion of the protocol five achieved CR, two PR and one was withdrawn from study at investigator preference. One patient has subsequently relapsed. Of the evaluable ten patients without a mediastinal presentation all achieved CR but three relapsed at 10, 13 and 18 months from diagnosis. Patients who achieved a PR only, relapsed or were withdrawn from study have been salvaged with MOPP or ChlVPP chemotherapy.

In childhood, there is growing evidence that all stages of Hodgkin's disease can be successfully treated with combination chemotherapy such as MOPP (Ekert & Waters, 1983; Ekert *et al.*, 1988) (mechlorethamine, oncovin, prednisone, procarbazine) or ChlVPP (Robinson *et al.*, 1984) (chlorambucil, vinblastine, prednisone, procarbazine) without irradiation. Irradiation in the treatment of early stage disease has been eliminated from these protocols because in the long term it causes growth retardation and its use as the only modality of treatment requires staging laparotomy and splenectomy in the majority of patients. Combined modality treatment has also been avoided because of concern with cumulative toxicity, including immunosuppression and second cancers (Meadows *et al.*, 1989). However, it is now well established that there are important long term toxicities of MOPP and ChlVPP of which the most serious are development of acute non-lymphocytic leukaemia (Meadows *et al.*, 1989) and infertility in males of all ages (Aubier *et al.*, 1989).

In an attempt to reduce the toxicity of treatment the Australian and New Zealand Children's Cancer Study Group (ANZCCSG) has piloted a potentially less toxic regimen utilising etoposide, vinblastine, Ara-C and cis-diaminodichloro-platinum (EVAP) (Wimmer *et al.*, 1987; Rybak *et al.*, 1990; Longo, 1990) as induction chemotherapy, followed by alternating courses of EVAP and doxorubicin hydrochloride (dox), vincristine, bleomycin (ABV). The use of etoposide and cis-diaminodichloro-platinum in the EVAP regimen was based on response rates of approximately 20% to etoposide and cis-diaminodichloro-platinum as single agents (O'Dwyer *et al.*, 1985; O'Reilly *et al.*, 1991; Corder *et al.*, 1979; Rossoff *et al.*, 1979; Cavilli, 1982). Ara-C was chosen because of its documented role in the treatment of non Hodgkins lymphoma and its previous use in the 'APE' combination chemotherapy regimen (Wimmer *et al.*, 1987) (cytosine arabinoside, cisplatin, etoposide).

We now report the results of the use of this chemotherapy regimen in 25 newly diagnosed children with all stages of Hodgkin's disease followed for a median of 37 months.

Correspondence: H. Ekert, Department of Clinical Haematology/Oncology, Royal Children's Hospital, Flemington Road, Parkville, Vic 3052, Australia.
Received 17 March 1992; and in revised form 13 August 1992.

### Patients and methods

Informed consent was obtained from all families and patients participating in the study. They were told that there were three main methods of treatment. One used irradiation and staging laparotomy for early stage disease or MOPP and irradiation for all stages. The second was MOPP chemotherapy alone while the third was the experimental protocol. The side effects of each modality of therapy were outlined. The patients and their family were told that relapses could be salvaged with MOPP and/or irradiation. All patients with biopsy proven Hodgkin's disease were eligible and all agreed to participate. At the time of biopsy, surgeons were requested not to attempt complete clearance of involved lymph nodes. Staging was clinical, using the Ann Arbor classification (Carbone *et al.*, 1971). All patients were further staged with chest and abdominal CT scans, double dose gallium scans and bone marrow trephines. Lymphography was not undertaken. In Table I are shown, the age, sex, histology and clinical stage of the patients entered into the study.

The chemotherapy regimen is summarised in Figure 1. In patients with measurable residual disease after biopsy, it consisted of vinblastine 4 mg $m^{-2}$, Ara-C 3 Gm $m^{-2}$, etoposide 120 mg $m^{-2}$ and cis-platin 40 mg $m^{-2}$, given on days 1 and 8 and repeated on days 29 and 36. Response to EVAP was assessed with chest or abdominal CT scans and double dose gallium scan, 3 weeks after completion of EVAP. The same imaging and nuclear medicine investigations were again repeated 3–4 weeks after completion of the full protocol.

In patients without measurable residual disease, vinblastine, Ara-C, etoposide and cisplatin were given on day 1 and vincristine 1.5 mg $m^{-2}$, dox 25 mg $m^{-2}$ and bleomycin 10 mg $m^{-2}$ on day 8. Three of these hybrid cycles of chemotherapy were given at 4 week intervals. Response to treatment was assessed with gallium scan 3 weeks after completion of therapy.

All patients with residual disease who achieved complete remission (CR) after two cycles of EVAP were given a further two cycles of hybrid EVAP/ABV for those with non-mediastinal presentation and four cycles for those with a mediastinal presentation and re-assessed as before. Patients with evidence of residual disease but no progression after two cycles of EVAP (PR) were given three further cycles of EVAP/ABV for non-mediastinal presentation and four for mediastinal presentation and reassessed 3–4 weeks after completion of chemotherapy. In assessing residual disease

**Table I** Clinical features, staging and response to treatment

| Pt. | Age | Sex | Sites of Pred. disease | Clinical stage | Hist. | Response to EVAP | Response end of protocol | Time to treatment failure (M) | DFS (M) |
|---|---|---|---|---|---|---|---|---|---|
| *No residual disease after biopsy* | | | | | | | | | |
| 1 | 10 | M | Cervical | IA | LP | NRD | CR | 36 + | 36 + |
| 2 | 3 | M | Cervical | IA | NS | NRD | CR | 46 + | 46 + |
| 3 | 5 | M | Cervical | IA | NS | NRD | NE | 1 (allergy) | 44 + |
| 4 | 13 | M | Cervical | IA | MC | NRD | CR | 42 + | 42 + |
| 5 | 13 | M | Cervical | IA | NS | NRD | CR | 31 + | 31 + |
| *Mediastinal enlargement* | | | | | | | | | |
| 6 | 14 | F | Mediastinal/cervical | IVA | NS | PR | CR | 34 + | 34 + |
| 7 | 14 | F | Mediastinal/abdominal | IIIA | NS | PR | PR | 7 | 46 + |
| 8 | 16 | M | Mediastinal/cervical | IIIB | NS | PR | PR | 6 | 44 + |
| 9 | 14 | F | Mediastinal/cervical | IIA | NS | CR | CR | 32 + | 32 + |
| 10 | 4 | M | Mediastinal/cervical | IIA | MC | CR | CR | 31 + | 31 + |
| 11 | 12 | M | Mediastinal | IA | NS | CR | CR | 24 | 47 + |
| 12 | 14 | F | Mediastinal/cervical | IIA | NS | CR | CR | 24 + | 24 + |
| 13 | 13 | M | Mediastinal | IA | MC | PR | NE | 3 (*) | 24 + |
| *Non-mediastinal lymph node involvement* | | | | | | | | | |
| 14 | 10 | M | Abdominal | IIB | MC | PR | CR | 35 + | 35 + |
| 15 | 7 | M | Cervical | IA | MC | CR | CR | 44 + | 44 + |
| 16 | 16 | F | Cervical/abdominal | IIIA | NS | CR | CR | 36 + | 36 + |
| 17 | 7 | M | Cervical | IA | LP | CR | CR | 18 | 39 + |
| 18 | 11 | M | Cervical | IA | NS | CR | CR | 37 + | 37 + |
| 19 | 14 | M | Cervical | IA | NS | PR | CR | 47 + | 47 + |
| 20 | 5 | F | Cervical | IA | LP | CR | CR | 40 + | 40 + |
| 21 | 8 | F | Cervical | IIA | LP | CR | CR | 10 | 38 + |
| 22 | 6 | M | Cervical | IA | MC | PR | CR | 13 | 30 + |
| 23 | 4 | M | Inguinal | IIA | NS | CR | CR | 5 (*) | 28 + |
| 24 | 3 | M | Cervical | IA | NS | NE | NE | – (anaph.) | 19 + |
| 25 | 14 | M | Cervical (lung) | IVA | NS | CR | CR | 24 + | 24 + |

N.S. nodular sclerosis; MC-mixed cellularity; LP-lymphocyte dominant; CR-complete remission; PR-partial remission (>50% reduction of tumour size); NRD-no residual disease; NE-not available; M-month; DFS-disease free survival; anaph-anaphylaxis; *-protocol violation.

after two cycles of EVAP mediastinal widening of any degree by chest radiography and CT scans was considered to represent persistence of Hodgkin's disease. However, if at the completion of treatment there was a decrease of mediastinal widening, but not a return to normal the patients were observed and only considered to have failed treatment if the mediastinum continued to widen, there was increased gallium uptake, or relapse at other sites.

Patients who had progressive disease after two cycles of EVAP; failed to achieve remission at the completion of the planned course of treatment; or who relapsed after treatment were changed to MOPP or ChlVPP and given at least three cycles.

The results of this study were analysed according to the recommendations of Dixon *et al.* (1987). The following end points are reported:

(i) *Survival*
Percent of patients surviving from entry into study until death or last known follow up time at least April 1991 for all patients.

(ii) *Treatment failure*
Percent of patients who have failed to respond, relapsed, died or were withdrawn for any reason from the time of entry on the protocol.

(iii) *Relapse*
Percent who achieved remission and subsequently relapsed at the completion of the full protocol of treatment.

### Results

The study commenced in June 1987 and ceased in June 1989. The results were assessed in April 1991. The demographic characteristics of all patients and the response to treatment with EVAP only and after completion of EVAP/ABV are shown in Table I.

**Figure 1** Treatment flow chart. EVAP consists of Etoposide 120 mg m$^{-2}$, V = vinblastine 4 mg m$^{-2}$, Ara-C 3 G m$^{-2}$ and cisplatin 40 mg m$^{-2}$. ABV consists of dox 25 mg m$^{-2}$, vincristine 1.5 mg m$^{-2}$ and bleomycin 10 mg m$^{-2}$.

### *Patients with residual disease after biopsy*

There were eight patients presenting with widening of the mediastinum $> \frac{1}{3}$ thoracic width. Their ages range from 4–16 years with a median of 14 and four were males. Clinical staging showed that two had IA, three 3IIA, one III A, one IIIB and one IVA disease.

There were 12 patients with non mediastinal presentation, nine male and three female. Their ages ranged from 3–16 years with a median of 8. Stage IA disease was present in seven, IIA in two, IIB in one, IIIA in one and IVA in one. The main presenting feature was cervical node enlargement in nine and inguinal in three.

At the completion of EVAP treatment four patients with mediastinal enlargement achieved CR and four PR. In the non mediastinal group one patient (No. 24) was withdrawn after the first cycle of EVAP because of an anaphylactic reaction to etoposide. Of the remaining 11, eight achieved CR and three PR after EVAP treatment. Thus of the 19 evaluable patients with residual disease after biopsy 12 achieved CR and seven PR. None had progressive disease.

At the completion of the protocol, of the eight patients with mediastinal enlargement five achieved CR, two remained in PR and one with PR (No. 13) was withdrawn because of investigator preference for ChlVPP therapy. The two patients with PR had significant mediastinal widening on chest radiography and CT scans, but no increased gallium uptake. They were observed for 8 weeks and reassessed with radiography, chest CT and gallium scans. One, (No. 7) had no further mediastinal widening but a positive gallium scan. The other, (No. 8) had an increase in mediastinal widening and a positive gallium scan. Both were treated with five courses of MOPP and remain in remission at 44 and 46 months from diagnosis.

Of the 12 patients with non mediastinal presentation, but with residual disease after biopsy 11 achieved CR at the completion of the protocol. One was withdrawn because of an anaphylactic reaction to etoposide after one cycle of EVAP. He was treated with ChlVAPP and remains in remission 19 months from diagnosis. Another patient (No. 23) achieved CR at the end of the protocol but his off treatment response could not be followed because of parental insistence that he be given an additional three courses of MOPP. He remains in remission.

Relapse of disease occurred in four patients. One had mediastinal disease (No. 11), achieved CR in response to EVAP and remained in CR at the completion of the protocol but relapsed at 24 months with Stage IVB disease. He was treated with MOPP and remains disease free 47 months from diagnosis.

Three patients with non mediastinal presentation (Nos. 17, 21, 22) subsequently relapsed at the site of previous disease at 10, 13 and 18 months from diagnosis. Two of these had achieved CR with EVAP and one PR. All three achieved CR with MOPP but one (No. 22) relapsed again and was treated with ABVD induction followed by autologous bone marrow transplantation with BCNU, Ara-C and Melphalan conditioning.

*Patients with no residual disease after diagnostic biopsy*

There were five patients in this category, they were all males and had Stage I cervical disease. One (No. 3) was withdrawn from the protocol with rash and fever considered to be due to Ara-C. He was treated with three cycles of MOPP and is in remission 44 months from diagnosis. The remaining four patients tolerated their treatment well and are in remission 31–46 months from diagnosis.

*Overall results*

Figure 2 shows that the overall survival is 100% with a median follow up time of 37 months from diagnosis. Relapse free survival could only be assessed in 19 patients and was 79%. Six patients were not evaluable because four were withdrawn from protocol and two failed to achieve CR. Treatment failure free survival was assessed in all patients and was 60%. It was 66% if the two patients withdrawn for physician and patient preference are excluded as treatment failures, but evaluated until the time of their withdrawal from study.

*Toxicity*

The regimen was well tolerated with only moderate emesis controllable with standard anti-emetics. Admissions to hospital were required for hematologic toxicity and fever and neutropenia following 15 of 132 EVAP courses. Of the 15 admissions platelet transfusions were required in three and packed red cell transfusions in three patients. Allergic reactions to etoposide and Ara-C each occurred in one patient. All patients received chemotherapy at time intervals specified in the protocol and none required dose reduction. There was no evidence of impairment of renal function as measured by serum creatinine levels but EDTA Cr clearances were not performed. Routine audiology was not performed. Fertility and cardiac function studies are planned for the future.

### Discussion

This is the first report of hybrid EVAP/ABV chemotherapy in newly diagnosed children with Hodgkin's disease. We elected to pilot this protocol for two reasons. First, because it may be potentially less toxic than MOPP, ChlVPP or ABVD, as high doses of alkylating agents and anthracyclines are avoided. This should reduce the incidence of infertility, second malignancies and cardiomyopathy. Second, we reasoned that because EVAP had been shown to be an

**Figure 2** Results of treatment in all patients.

effective remission induction combination in previously treated patients with Hodgkin's disease (Wimmer et al., 1987; Rybak et al., 1990; Longo et al., 1990) it should also be effective in newly diagnosed patients. Furthermore, the use of a hybrid EVAP/ABV combination permitted reduction of the cumulative dose of anthracycline to a non-cardiotoxic dose of no more than 100 mg $m^{-2}$ and limited the dose of cisplatin to no more than 320 mg $m^{-2}$. These dose levels of cisplatin have only a low incidence of nephrotoxicity and hearing disturbances. We considered the hybrid combination sufficiently intensive to eliminate DTIC from the ABVD regimen.

In order to assess the effectiveness of EVAP in remission induction the protocol was constructed to allow investigation of response to two cycles of EVAP alone. Our results showed that two cycles of EVAP induced CR in 12 and PR in seven of the 19 evaluable patients. (Five no evaluable disease after biopsy, one anaphylactic reaction to etoposide). Four of the seven patients in PR presented with major mediastinal disease which at the time of reassessment had reduced in size, but still showed mediastinal widening and reduced but persistent gallium uptake.

It is possible that these patients may have achieved CR had the investigations been repeated some weeks later but we did not consider this to be in the patient's best interest as it would have meant a delay in subsequent chemotherapy.

The hybrid EVAP/ABV chemotherapy was effective in eliminating microscopic disease in the four patients who had no residual disease after biopsy and tolerated this regimen. It was also effective in the seven patients who achieved PR at the completion of two cycles of EVAP as only two failed to achieve CR at the completion of the protocol. It is likely that the ABV component of the hybrid protocol was of major importance in these patients progress from PR to CR.

Unlike the results that we previously reported with MOPP or Ch1VPP (Ekert & Water, 1983; Ekert et al., 1988) there was a significant incidence of relapse with four of 19 evaluable patients who achieved CR relapsing at sites of previous disease. There was also a high incidence of treatment failures. This was largely due to side effects of Ara-C and etoposide and withdrawal of patients by investigators despite the fact that the patients were in CR at the time of withdrawal. These are not uncommon events in a pilot study using an unconventional treatment regimen.

The purpose of this study was to pilot a potentially effective and less toxic chemotherapy regimen. Our results show that it was effective in achieving a lasting disease free status in 60% of patients including four of eight with major mediastinal disease. All patients who failed on the protocol could be salvaged with more intensive chemotherapy, such as MOPP. The duration of follow-up is still too short to be certain that late relapses will not occur in these patients. While the EVAP/ABV hybrid cannot be considered as an optimum low intensity, potentially less toxic therapy for newly diagnosed patients, it points the way for further pilot studies by demonstrating partial effectiveness and the ability to salvage at least in the short term chemotherapy failures with alternate chemotherapy.

### References

AUBIER, F., FLAMANT, F., BRAUNER, J.M., COILLAUD, J.M., CHAUSSAIN, J.M. & LEMERLE, J. (1989). Male gonadal function after chemotherapy for solid tumours in childhood. *J. Clin. Oncol.*, **7**, 304–309.

CARBONE, P.P., KAPLAN, H.S., MUSSHOFF, K., SMITHERS, D.W. & TUBIANA, M. (1971). Report of the committee on Hodgkin's disease staging classification. *Cancer Res.*, **31**, 1860–1861.

CAVILLI, F. (1982). VP16-213 (Etoposide); a critical review of its activity. *Cancer Chemother. Pharmacol.*, **7**, 81–85.

CORDER, M.P., ELLIOTT, T.E., MAGUIRE, L.C., LEIMERT, J.T., PANTHER, S.K. & LACHENBRUCH, P.A. (1979). Phase II study of Cis-dichlorodiamine platinum in lymphomas. A Southwest Oncology Group Study. *Cancer Treat. Rep.*, **63**, 763–766.

DIXON, D.O., MCLAUGHLIN, P., HAGEMEISTER, F.B., FREIREICH, E.J., FULLER, L.M., CABANILLAS, F.F. & GEHAN, E.A. (1987). Reporting outcomes in Hodgkin's disease and lymphoma. *J. Clin. Oncol.*, **5**, 1670–1672.

EKERT, H. & WATERS, K.D. (1983). Results of treatment of 18 children with Hodgkin's disease with MOPP chemotherapy as the only treatment modality. *Med. Pediatr. Oncol.*, **11**, 322–326.

EKERT, H., WATERS, K.D., SMITH, P.J., TOOGOOD, I. & MAUGHER, D. (1988). Treatment with MOPP and Ch1VPP chemotherapy only for all stages of childhood Hodgkin's disease. *J. Clin. Oncol.*, **6**, 1845–1850.

LONGO, D.L. (1990). The use of chemotherapy in the treatment of Hodgkin's disease. *Seminars in Oncol.*, **17**, 716–735.

MEADOWS, A.T., OBRINGER, A.C., MARRERO, O., OBERLIN, O., ROBISON, L., FOSSATI-BELLANI, F., GREEN, D., VOUTE, P.A., MORRIS-JONES, P., GREENBERG, M., BAUM, E. & RUYMANN, F. (1989). Second malignant neoplasms following childhood Hodgkin's disease: treatment and splenectomy as risk factors. *Med. Pediatric. Oncol.*, **17**, 477–484.

O'DWYER, P.J., LEYLAND-JONES, B., FLOUSO, M.T., MARSONI, S. & WITTES, R.E. (1985). Etoposide (VP-16-213). Current status of an active anti cancer drug. *N E J M*, **312**, 692–700.

O'REILLY, S.E., KLIMO, P. & CONNORS, J.M. (1991). The evolving role of etoposide in the management of lymphomas and Hodgkin's disease. *Cancer*, **67** (Suppl 1), 271–280.

ROBINSON, B., KINGSTON, J., COSTA, R.N., MALPAS, J.S., BARRETT, A. & MCELWAIN, T.J. (1984). Chemotherapy and irradiation in childhood Hodgkin's disease. *Arch. Dis. Child.*, **59**, 1162–1167.

ROSSOFF, A.H., COTTMAN, C.A., JONES, S.E. & TALLEY, R.W. (1979). Phase II evaluation of cis-dichloradiamine platinum in Stage IVB Hodgkin's disease. *Cancer Treat. Rep.*, **63**, 1605–1608.

RYBAK, M.E., MCCARROLL, K., KAPLAN, R.J., PROPERT, K.J., BUDMAN, D.R. & GOTTLIEB, A.J. (1990). Phase II trial of etoposide and cis diaminodichloro-platinum in patients with refractory and relapsed Hodgkin's disease: Cancer and Leukemia Group B (CALGB) study 8353. *Med. Pediatr. Oncol.*, **18**, 177–180.

WIMMER, R., WEINER, M., STRAUSS, L., LEVENTHAL, B., ADAIR, S., KLETZELA, M. & HARTMANN, G. (1987). Treatment of paediatric patients for relapsed Hodgkin's disease (HD) with cytosine arabinoside (A) Cisplatin (P) and etoposide (E). *Proc. Am. Soc. Clin. Oncol.*, **6**, A753, 1987.